# Urate-lowering Therapy and Chronic Kidney Disease Development in Patients with Gout

**DOI:** 10.7150/ijms.59698

**Published:** 2021-04-29

**Authors:** Fu-Shun Yen, James Cheng-Chung Wei, Chia-Ling Chang, Chen-Chang Yang, Chih-Cheng Hsu, Chii-Min Hwu

**Affiliations:** 1 Dr. Yen's Clinic, Taoyuan City, Taiwan.; 2 Institute of Medicine, Chung Shan Medical University, Taichung, Taiwan.; 3 Department of Medicine, Chung Shan Medical University Hospital, Taichung, Taiwan.; 4 Graduate Institute of Integrated Medicine, China Medical University, Taichung, Taiwan.; 5 Management Office for Health Data, China Medical University Hospital, Taichung City, Taiwan.; 6 Graduate Institute of Clinical Medical Science, College of Medicine, China Medical University, Taichung City, Taiwan.; 7 Institute of Environmental & Occupational Health Sciences, School of Medicine, National Yang-Ming University, Taipei, Taiwan.; 8 Institute of Public Health, School of Medicine, National Yang-Ming University, Taipei, Taiwan.; 9 Division of Clinical Toxicology & Occupational Medicine, Department of Medicine, Taipei Veterans General Hospital, Taipei, Taiwan.; 10 Institute of Population Health Sciences, National Health Research Institutes, Zhunan, Miaoli, Taiwan.; 11 Department of Health Services Administration, China Medical University, Taichung, Taiwan.; 12 Department of Family Medicine, Min-Sheng General Hospital, Taoyuan, Taiwan.; 13 Faculty of Medicine, National Yang-Ming University School of Medicine, Taipei, Taiwan.; 14 Section of Endocrinology and Metabolism, Department of Medicine, Taipei Veterans General Hospital, Taipei, Taiwan.

**Keywords:** gout, urate-lowering therapy, chronic kidney disease, diabetes mellitus, hypertension

## Abstract

**Objectives:** Chronic kidney disease (CKD) has emerged as a global health concern. Many studies have identified an association between hyperuricemia and CKD, and some studies have revealed that urate-lowering therapy (ULT) can attenuate CKD progression. However, only a few studies have explored the role of ULT in the prevention of new onset CKD.

**Methods:** To compare the risk of incident CKD between users and nonusers of ULT in patients with gout, we conducted a 13-year population-based retrospective cohort study. Overall incidence of CKD was compared between 7126 ULT users and 7126 matched ULT nonusers.

**Results:** The CKD incidence rate for both the users and nonusers of ULT was 1.7 per 100 person-years, after adjusting for sex, age, region of residence, comorbidities, and medications used. No significant difference in CKD risk (adjusted hazard ratio [aHR]: 0.97; 95% confidence interval [CI]: 0.88-1.07) was noted between the ULT users and nonusers. In the subgroup of patients with diabetes mellitus (DM) and without hypertension (HT), ULT tended to be associated with lower risk of incident CKD (aHR: 0.52; 0.95% CI: 0.28-0.97). Compared with the risk of new onset CKD in patients receiving xanthine oxidase inhibitors, those receiving uricosuric agents seemed to have a lower risk of developing CKD (aHR: 0.81, 95% CI: 0.67-0.99).

**Conclusion:** This population-based cohort study indicated that ULT is not associated with lower risk of CKD development. However, in the subgroup of patients with DM and without HT, ULT is associated with significantly lower risk of incident CKD.

## Introduction

Chronic kidney disease (CKD) has emerged as a global health concern, not only because it could develop an irreversible health problem such as end-stage renal disease or renal replacement, but because it could provoke a high mortality rate 1. In the United States, the CKD prevalence increased from 10% in 1988 to 14.8% in 2016 2; the number of patients with end-stage renal disease increased more than 3 times from 199,548 in 1991 to 726,331 in 2016 3. In Taiwan, the overall prevalence of CKD increased from 2.0% in 1996 to 11.9% in 2006 4; the incidence and prevalence rates of dialysis were among the highest in the world for more than 10 years 5. In a study, patients with end-stage renal disease over the age of 65 years had a 3.7-11.7 times higher risk of mortality than an age-matched general Medicare population 3. To determine the risk factors for CKD development or progression and how to treat them is a compelling public health concern. In fact, only few proven effective therapies for CKD are available.

CKD seems to develop easily in patients with gout, which may be due to the deposition of urate crystals and the generation of gouty nephropathy with pathology of glomerulosclerosis, interstitial fibrosis, and renal arteriolosclerosis 6, although some variants such as familial juvenile hyperuricemia share similar histological features except rare urate deposition 7. Hyperuricemia may be involved in CKD development independently. A meta-analysis demonstrated that hyperuricemia could increase the risk of CKD 8. Most patients with hyperuricemia also have metabolic diseases, which can easily result in CKD 9. Therefore, whether hyperuricemia is a marker or risk factor for CKD is still subject to debate.

Urate-lowering therapy (ULT), which includes xanthine oxidase (XO) inhibitors, uricosuric agents, and uricases, can effectively decrease the serum uric acid level 10. If using ULT to decrease serum uric acid can attenuate CKD development or progression, then it might indicate that serum uric acid was the risk factor for CKD. A meta-analysis on ULT use for improving renal outcomes in CKD patients revealed that lowering the urate level had beneficial renal effects 11. However, only a few studies investigating association between ULT use and new onset CKD have been conducted 12-14. Therefore, we conducted this retrospective cohort study to determine the relationship between ULT use and the risk of incident CKD.

## Materials and Methods

### Patients and data source

We used the Longitudinal Health Insurance Database 2000, which comprises 1 million randomly selected participants from Taiwan's National Health Insurance Database (NHIRD), to explore the association of ULT and CKD development in patients with gout. The NHIRD comprises the outpatient and inpatient healthcare data of the National Health Insurance (NHI) program. NHI has been implemented since 1995 and includes more than 99% of the residents of Taiwan 15. This dataset includes the birthdate, sex, medical procedures, prescriptions, and disease diagnoses according to the International Classification of Diseases, Ninth Revision, Clinical Modifications (ICD-9-CM), of the insured. Information that can be used to identify patients or caregivers is encrypted before data release. Our study was approved by the Research Ethics Committee of China Medical University and Hospital (CMUH-104-REC2-115). We were granted a waiver of the informed consent.

### Study design

The study population included patients with ≥2 diagnoses of gout (ICD-9-CM codes: 274.00-274.03, 274.8-274.9) in outpatient claims or ≥1 discharge diagnose in inpatient claims and the use of colchicine. We enrolled 12,845 patients who received ULT [allopurinol (M04AA01), febuxostat (M04AA03), benzbromarone (M04AB03), sulfinpyrazone (M04AB02), and probenecid (M04AB01)] from January 2000 to December 2012 as the user cohort. The first date of claims for ULT use was defined as the index date. We excluded participants with an age less than 20 years or more than 80 years, with preexisting CKD before the index date or a diagnosis of CKD within 180 days after the index date, with preexisting cancer before the index date, or with missing data for sex, age, or region of residence. Participants for the comparison cohort were selected from patients who did not receive ULT, using the same exclusion criteria as the user cohort. This was followed by 1:1 propensity score matching by age, sex, index year, Charlson comorbidity index (CCI) 16, region of residence, comorbidities, and medications. The index date for the comparison cohort was a randomly assigned date within the follow-up period after the diagnosis of gout.

### Primary outcome

The primary outcome of this study was new onset CKD, which was diagnosed by two or more outpatient claims (ICD-9-CM codes: 403, 404, 580-589, V42.0, V45.1, V56.X, and A350) or at least one inpatient claim. The algorithm using ICD-9 for defining CKD was validated in a previous study with acceptable accuracy 17. The follow-up person-years were calculated from enrollment to CKD development, death, withdrawal from the insurance program, or the end of the study (December 31, 2013), whichever came first. We did a series of stratified subgroup analyses to see whether ULT have different impact on CKD development in persons with different age, comorbidities, and medications use. In order to know whether the various urate-lowering drugs have different effects on CKD development, we also observed the risks of incident CKD in four categories of patients [non-users, XO inhibitors (use XO inhibitors only), uricosuric agents (use uricosuric agents only), both (patients who have used XO inhibitors and uricosuric agents)]. We used non-users as a reference at first; and then we used the most studied XO inhibitors as a reference at the second time.

### Demographic information, comorbidity and medications

The baseline characteristics of patients retrieved for analysis were sex, age, region of residence, comorbidities, and prescribed medications. Comorbid diseases included in this study were hypertension (HT; ICD-9-CM codes: 401-405, and A26), diabetes mellitus (DM; ICD-9-CM codes: 250.x0, 250.x2, and A181), hyperlipidemia (ICD-9-CM codes: 272, 278, and A189), coronary artery disease (ICD-9-CM codes: 410-414), cerebrovascular diseases (ICD-9-CM codes: 362.34 and 430.x-438.x), heart failure (ICD-9-CM code: 428), peripheral vascular diseases (ICD-9-CM codes: 093.0, 437.3, 440.x, 441.x, 443.1-443.9, 47.1, 557.1, 557.9, and V43.4), rheumatologic diseases (ICD-9-CM codes: 446.5, 710.0-710.4, 714.0-714.2, 714.8, and 725.x), alcohol-related diseases (ICD-9-CM codes: 291.x, 303.x, 305.0, 357.5, 425.5, 535.3, 571.0-571.3, 980.0, and E947.3), and obesity (ICD-9-CM codes: 278, 783.1, and V77.8). To increase the validity of the diagnoses in the administrative dataset for these comorbidities, only patients with ≥2 outpatient or ≥1 inpatient claims were included. Other comorbid profiles of the patients were quantified using CCI. The comorbidities and CCI were calculated based on the status of the patient 1 year before the index date. The use of medications other than ULT and colchicine were assessed, including angiotensin-converting enzyme inhibitors (ACEIs), angiotensin II receptor blockers (ARBs), β-blockers, calcium-channel blockers, diuretics, potassium-sparing diuretics, metformin, sulphonylureas, insulin, statins, and aspirin.

### Statistical analysis

Distribution of sex, age, region of residence, comorbidities, CCI, and medications in the user and comparison cohorts are expressed as numbers and percentages. The differences between ULT users and nonusers were analyzed by chi-square and *t* tests. We used propensity score match to balance the potential confounders of ULT users and non-users to augment their comparability. Although some unmeasured confounding factors may still exist disproportionally in these two study groups, propensity score match can optimally balance distributions of measured covariates as much as randomized study designs do. We estimated the propensity score for every patient by a nonparsimonious multivariable logistic regression by assigning receipt of ULT as the dependent variable. We incorporated 32 clinically relevant covariates into our analysis as independent variables (including baseline characteristics, comorbidities, and medications use). We applied the greedy nearest-neighbor algorithm to construct matched pairs, assuming that the standardized mean difference ≤ 0.10 as a negligible difference between the two groups 18. The incidence rate (IR) of incident CKD was defined as the number of events divided by person-years. The Cox proportional hazard model was applied to estimate hazard ratios (HRs) and 95% confidence intervals (CIs) of ULT use-related CKD development. Cumulative incidence curves of CKD for the user and comparison cohorts were computed using the Kaplan-Meier method, and the differences were analyzed using the log-rank test. All statistical analyses were performed using STATA/SE version 14.0 (STATA Corp., College Station, TX). Statistical significance was determined using two-tailed tests. To account for effects of multiple comparisons conducted in this study, we applied a Bonferroni correction to set the significance level at a *p* < 0.002.

## Results

**Figure 1:**
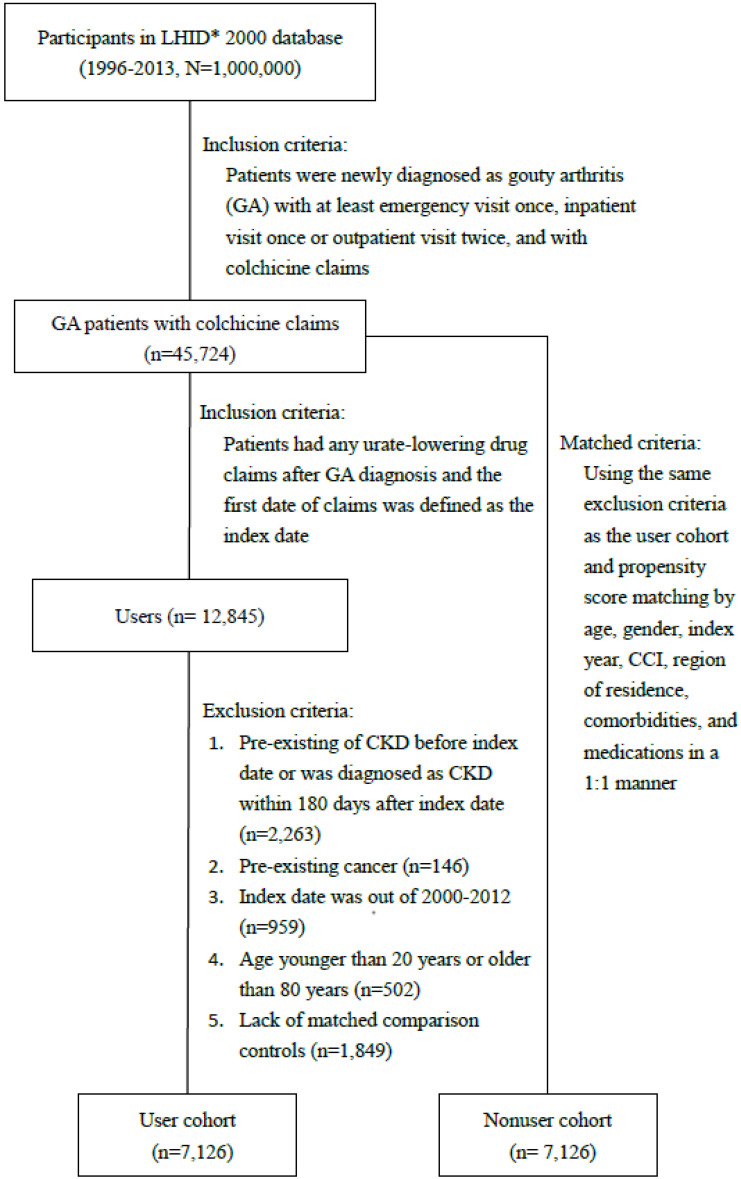
Flow chart of the process for establishing the urate-lowering therapy and control cohorts. LHID, Longitudinal Health Insurance Database.

Figure 1 depicts the flow chart for establishing the ULT user and control cohorts using data derived from the NHIRD. The baseline characteristics of un-matched patients using and non-using ULT are listed in Table 1 to provide information about the original condition of the study and control cohorts. After propensity score matching, the number of patients with and without ULT use in each cohort was 7126 (Table 1). The variables, sex, age, region of residence, comorbidities, and medications use, were balanced with a standardized mean difference ≤0.10. The mean (standard deviation, SD) ages in the user and nonuser cohorts were 48 (15) years and 47.9 (14.1) years, respectively. The means (SD) of the follow-up time in the user and nonuser cohorts were 6.4 (3) years and 6.4 (3.4) years, respectively. The mean period between from gout diagnosis to the beginning of urate lowering therapy was 1.10 (2.04) years.

Table 2 reveals that female, older age, hypertension, DM, coronary artery disease, cerebrovascular disease, heart failure, peripheral vascular disease, rheumatologic disease and higher Charlson Comorbidity Index are associated with higher risk of CKD development.

Table 3 presents that the IR of new onset CKD in both the ULT user and nonuser cohorts was 1.7 per 100 person-years. Compared with the nonusers, the ULT users had a 0.97-fold adjusted HR for CKD development (*p* = 0.581). Regarding CKD development, compared with the nonusers, the users of XO inhibitors had an adjusted HR of 1.07 (95% CI: 0.93-1.22), and the users of uricosuric agents had an adjusted HR of 0.93 (95% CI: 0.83-1.04). Table 3 also shows the adjusted HRs of CKD development for the ULT users in different subgroups. For example, the adjusted HR is 0.98 (95% CI: 0.88-1.09) for those without HT and DM; 1.07 (95% CI: 0.70-1.63) for those with HT but no DM; 1.52 (95% CI: 0.20-11.29) for those with HT and DM; 0.52 (95% CI: 0.28-0.97) for those with DM but no HT.

**Figure 2:**
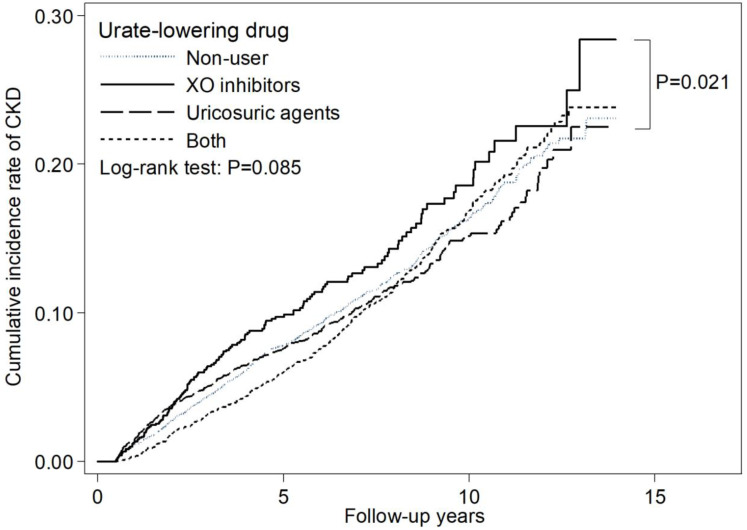
Cumulative incidence curve of CKD by urate-lowering therapy.

We used the most studied XO inhibitors as a reference, uricosuric agent users seemed to have a lower adjusted HR of 0.81 (95% CI: 0.67-0.99) for incident CKD as compared with XO inhibitor users (Table 4). Figure 2 depicts the cumulative incidence of CKD in patients receiving XO inhibitors, uricosuric agents, both, and those not receiving any ULT with the Kaplan-Meier curve, and the *p* value of the log-rank test was found to be 0.085. There is no statistically significant difference among these four groups. However, XO inhibitor users seemed to have a higher cumulative incidence of CKD compared with uricosuric agent users.

Compared with ULT nonusers, ULT users were not associated with significantly different risk of incident CKD; but in the subgroup of patients with DM and without HT, ULT users tended to be associated with lower risk of incident CKD. Among the four categories of ULT using, uricosuric users seemed to have a lower risk of incident CKD as compared with XO inhibitor users by multivariate analysis and log-rank test. However, they do not reach a statistical significance after applying the Bonferroni correction to account for the effects caused by multiple comparisons.

## Discussion

After propensity score matching for sex, age, region of residency, comorbidities, and medications use, it was found that ULT users were not associated with lower risk of CKD development. Although not statistically significant, in the subgroup of patients with DM and without HT, ULT users tended to be associated with lower risk of incident CKD; the four categories of ULT using, uricosuric users seemed to have a lower risk of incident CKD as compared with XO inhibitor users by multivariate analysis and log-rank test.

Previous studies have indicated a close association between hyperuricemia and CKD. Sonoda et al. conducted a clinical study with a large case series and revealed that hyperuricemia could be a predictor of renal dysfunction 19. A meta-analysis of 13 observational studies performed by Li et al. revealed that elevated uric acid levels increased the risk of new onset CKD 8. Clinical and animal studies have demonstrated that hyperuricemia can induce HT and renal injury by stimulating the renin-angiotensin system, suppressing neuronal nitric oxide synthase, lowering endothelial nitric oxide levels, and enhancing endothelial dysfunction in the kidney 20. Soluble uric acid can stimulate vascular smooth muscle cell proliferation through the mitogen-activated protein kinase pathway, leading to afferent arteriolopathy and the impairment of renal autoregulation 21. Uric acid can promote vascular smooth muscle cells to produce hydrogen peroxide and 8-isoprostane 22, increasing oxidative-stress-generated pro-oxidative effect. Uric acid can also activate vascular smooth muscle cells and endothelial cells to produce cytokines and c-reactive protein 23,24 and this proinflammatory effect might intensify the proatherogenic properties of uric acid.

Kanji et al. performed a systemic review and meta-analysis of 19 randomized controlled trials on patients with stage 3-5 CKD and lowered urate levels. The study revealed that allopurinol significantly reduces serum uric acid level and retards CKD progression 11. Pisano et al. conducted a systemic review that revealed XO inhibitors could reduce the risk of renal failure in patients with CKD with or without gout 25; but no significant improvement in proteinuria and serum creatinine levels was observed compared with the control group. Doria et al. conducted a randomized double-blind trial of patients with type 1 diabetes, serum urate levels of at least 4.5 mg per deciliter and an eGFR of 40.0 to 99.9 ml per minute per 1.73 m^2^ of body-surface area, and recently reported that no clinically meaningful benefits of uric acid reduction with allopurinol on renal outcomes in type 1 diabetes patients with early-to-moderate diabetic kidney disease 26. Simultaneously, Badve et al. also reported a randomized control trial of adults with stage 3 or 4 chronic kidney disease and no history of gout, and disclosed that urate lowering therapy with allopurinol did not slow the decline in eGFR as compared with placebo in patients with chronic kidney disease and higher risk of progression 27. Because most studies included patients with CKD, whether ULT could prevent the development of new onset CKD could not be determined.

Few studies on ULT use and CKD development are available. Kanbay et al. conducted a prospective study in which 48 hyperuricemic and 21 normouricemic patients with normal renal function were followed for 3 months. The hyperuricemic patients were given allopurinol 300 mg per day for 3 months. The results showed significant improvement in eGFR at 3 months with allopurinol 12. Liu et al. conducted an open label randomized trial in 176 patients with diabetes and asymptomatic hyperuricemia who were randomized to receive allopurinol or conventional treatment. At the end of 3rd year, GFR was better preserved in the group that received allopurinol 13. Vargas-Santos et al. did a propensity score analysis to assess the association of allopurinol use in gout with the risk of developing chronic kidney disease stage 3 or higher. The results showed allopurinol initiation of at least 300 mg per day was associated with a lower risk of renal function deterioration 14. These 3 studies used allopurinol in patients with hyperuricemia or gout with normal renal functions and disclosed that allopurinol may attenuate CKD development. Our study revealed that ULT use in patients with gout was not associated with lower risk of CKD development, whereas in the patient with DM and without HT, ULT use may be able to mitigate the risk of new onset CKD. The reasons for the difference between our study and above mentioned 3 studies might be: 1. We used XO inhibitors and uricosuric agents as ULT use, whereas only XO inhibitor of allopurinol was used in above 3 studies. 2. All our patients had gout and those in Kanbay's and Liu's patients were asymptomatic hyperuricemia. 3. The number of patients, patient population and study design were different among these studies.

The plausible explanations for the results of our study are as follows: in patients without DM and HT, the magnitude and duration of ULT use for attenuating CKD development might not have been enough 8,28. It was believed that after HT occurrence, the structural damage of kidneys becomes the main mediator for CKD development and lowering serum uric acid level would not influence the development of CKD 29. In patients with DM and without HT, timely use of ULT may have improved endothelial damage and maintained glomerulus autoregulation 13. These results indicate that a large series study of patients with DM and without HT is required to confirm the possibility of using ULT for preventing CKD development.

We compared the renoprotective effects of ULT and uricosuric agents and found that uricosuric agents were probably more beneficial for preventing CKD development. This might indicate that for preventing renal function decline, enhancing uric acid excretion might be more useful than using XO inhibitors with their antioxidant effect. Uricosuric agents can reduce the activity of urate transporter 1, and consequently, decrease the active reabsorption of uric acid in the renal proximal tubule 30, which may be able to diminish energy consumption due to uric acid reabsorption and attenuate renal function declining. In addition, the rare severe hypersensitivity reaction of allopurinol and the increased cardiovascular mortality due to febuxostat 31 should be considered before administration of XO inhibitors.

This study has some advantages and limitations. First, this was a large population-based study, in which data on consecutive patients were obtained from the NHIRD to avoid selection bias. Thus, observations of this study may be generalizable to relevant medical settings. However, almost all patients were Chinese; therefore, the results may not be applicable to other ethnicities. Second, we included patients with HT, DM, and other comorbidities because we wanted to explore the possible associations between ULT use and the risk of incident CKD in real-world settings. Notably, in the subgroup with DM and without HT, ULT use seemed to be associated with lower risk of new onset CKD. Third, we did not have information regarding the smoking and alcohol drinking habits, physical activity, and body mass index of the patients, which may influence the outcome. In addition, the results of measured blood pressure levels and biochemical blood tests such as tests for uric acid, glucose, hemoglobin A1C, creatinine levels and eGFR, which could provide more detailed information of the patient's clinical condition, were not available. Some patients might have lower uric acid levels with less gouty attack and no use of ULT. However, 32 important variables were maximally balanced through propensity score matching to minimize possible confounding effects. Fourth, some patients with high serum uric acid levels, but without gout attacks, may not be included for observation. Some patients may have non-gouty arthritis, but they are mistakenly selected for study because of no serum uric acid values available. Fifth, in many instances, the administrative data did not have records regarding urinary albumin excretion rate, proteinuria, or other hints of early-stage CKD, which may have led to some underestimate of CKD. Finally, this study was a retrospective cohort study; hence, some inevitable biases might exist. Thus, a randomized control study is required to verify the key findings of this study.

In conclusion, in this population-based cohort study, ULT use in patients with gout was not associated with lower risk of CKD development. However, in the subgroup of patients with DM and without HT, ULT tended to be associated with lower risk of incident CKD. Comparing the effects of ULT use on CKD development, uricosuric agents seemed to have association with lower risk of incident CKD than XO inhibitors.

## Figures and Tables

**Table 1:** Baseline characteristics of the urate-lowering therapy users and nonusers

	Original (un-matched) Cohort			Matched Cohort		
	Urate-lowering Therapy		*P* value^*^	Urate-lowering Therapy		SMD^**^
	No (N = 15512)	Yes (N = 8975)		No (N = 7126)	Yes (N = 7126)	
	N (%)	N (%)		N (%)	N (%)	
**Gender**			<0.001			0.009
Female	2511 (16.2)	1220 (13.6)		1010 (14.2)	1032 (14.5)	
Male	13001 (83.8)	7755 (86.4)		6116 (85.8)	6094 (85.5)	
**Age, years**			<0.001			
20-39	3579 (23.1)	3477 (38.7)		2265 (31.8)	2378 (33.4)	0.034
40-59	7412 (47.8)	3750 (41.8)		3399 (47.7)	3094 (43.4)	0.086
60-79	4521 (29.2)	1748 (19.5)		1462 (20.5)	1654 (23.2)	0.065
Mean (SD)	51.6 (14.3)	46.0 (15)	<0.001	47.9 (14.1)	48.0 (15)	
**Region of residence**			<0.001			
North	7226 (46.6)	4208 (46.9)		3287 (46.1)	3330 (46.7)	0.012
Central	3674 (23.7)	1882 (21.0)		1697 (23.8)	1507 (21.2)	0.064
South	4007 (25.8)	2550 (28.4)		1845 (25.9)	2023 (28.4)	0.056
East	564 (3.6)	310 (3.5)		278 (3.9)	251 (3.5)	0.020
Others	41 (0.3)	24 (0.3)		19 (0.3)	15 (0.2)	0.012
**Co-morbidity**						
Hypertension	640 (4.1)	330 (3.7)	0.083	288 (4.0)	283 (4.0)	0.004
DM	415 (2.7)	154 (1.7)	<0.001	131 (1.8)	140 (2.0)	0.009
Hyperlipidemia	825 (5.3)	393 (4.4)	0.001	346 (4.9)	349 (4.9)	0.002
CAD	414 (2.7)	176 (2.0)	<0.001	169 (2.4)	158 (2.2)	0.010
CVD	201 (1.3)	85 (1.0)	0.014	84 (1.2)	74 (1.0)	0.013
Heart failure	112 (0.7)	42 (0.5)	0.015	35 (0.5)	39 (0.6)	0.008
PVD	97 (0.6)	38 (0.4)	0.04	34 (0.5)	36 (0.5)	0.004
RD	123 (0.8)	68 (0.8)	0.762	51 (0.7)	59 (0.8)	0.013
ARD	113 (0.7)	50 (0.6)	0.112	39 (0.6)	45 (0.6)	0.011
Obesity	42 (0.3)	27 (0.3)	0.669	21 (0.3)	21 (0.3)	0.000
**CCI**			<0.001			
0	12974 (83.6)	8084 (90.1)		6238 (87.5)	6303 (88.5)	0.028
1	1750 (11.3)	643 (7.2)		666 (9.4)	578 (8.1)	0.044
≥2	788 (5.1)	248 (2.8)		222 (3.1)	245 (3.4)	0.018
**Medications**						
ACEI/ARBs	2467 (15.9)	1013 (11.3)	<0.001	912 (12.8)	924 (13.0)	0.005
β-Blocker	3114 (20.1)	1290 (14.4)	<0.001	1185 (16.6)	1165 (16.4)	0.008
CCB	3323 (21.4)	1342 (15.0)	<0.001	1229 (17.3)	1226 (17.2)	0.001
Diuretics	1837 (11.8)	849 (9.5)	<0.001	764 (10.7)	757 (10.6)	0.003
PSD	165 (1.1)	62 (0.7)	0.003	57 (0.8)	54 (0.8)	0.005
Metformin	780 (5.0)	309 (3.4)	<0.001	275 (3.9)	287 (4.0)	0.009
Sulfonylurea	891 (5.7)	330 (3.7)	<0.001	306 (4.3)	308 (4.3)	0.001
Insulin	160 (1.0)	62 (0.7)	0.007	59 (0.8)	56 (0.8)	0.005
Statin	1085 (7.0)	284 (3.2)	<0.001	304 (4.3)	280 (3.9)	0.017
Aspirin	1538 (9.9)	601 (6.7)	<0.001	576 (8.1)	551 (7.7)	0.013
Follow-up year				6.4 (3.4)	6.4 (3)	

SD: standard deviation; DM: diabetes mellitus; CAD: coronary artery disease; CVD: cerebrovascular disease; PVD: peripheral vascular disease; RD: rheumatologic disease; ARD: alcohol-related disease; CCI: Charlson comorbidity index; ACEI: angiotensin-converting-enzyme inhibitor; ARBs: angiotensin-receptor blockers; CCB: calcium channel blocker; PSD: potassium sparing diuretic. *Tested by chi-square or t test. **SMD, standardized mean difference, ≤0.10 indicates a negligible difference between the two cohorts.

**Table 2:** The incidence rates and risk factors for CKD development

	No. CKD	Person-years	IR	Crude HR (95% CI)	*p*-value
**Urate-lowering therapy**					
Non-user	782	45,490	1.7	Reference	
User	794	45,799	1.7	1.00 (0.91-1.11)	0.945
**Gender**					
Female	274	13,016	2.1	Reference	
Male	1302	78,273	1.7	0.79 (0.69-0.90)	<0.001
**Age, year**					
20-39	238	29,930	0.8	Reference	
40-59	653	42,253	1.5	1.94 (1.67-2.25)	<0.001
60-79	685	19,106	3.6	4.49 (3.87-5.20)	<0.001
**Co-morbidity**					
Hypertension	101	3,678	2.7	1.63 (1.33-1.99)	<0.001
DM	63	1,735	3.6	2.14 (1.66-2.75)	<0.001
Hyperlipidemia	88	5,188	1.7	0.95 (0.77-1.18)	0.64
CAD	66	2,203	3.0	1.74 (1.36-2.22)	<0.001
CVD	38	1,014	3.7	2.19 (1.59-3.02)	<0.001
Heart failure	17	406	4.2	2.48 (1.54-4.00)	<0.001
PVD	17	472	3.6	2.06 (1.28-3.33)	0.003
RD	23	740	3.1	1.79 (1.18-2.70)	0.006
ARD	10	546	1.8	1.08 (0.58-2.01)	0.805
Obesity	5	275	1.8	1.06 (0.44-2.54)	0.903
**CCI**					
0	1232	81,307	1.5	Reference	
1	238	7,521	3.2	2.11 (1.83-2.42)	<0.001
2	106	2,460	4.3	2.93 (2.40-3.58)	<0.001

IR, incidence rate (per 100 person-years); HR, hazard ratio; DM: diabetes mellitus; CAD: coronary artery disease; CVD: cerebrovascular diseases; PVD: peripheral vascular diseases; RD: rheumatologic diseases; ARD: Alcohol-related diseases; CCI: Charlson Comorbidity Index.

**Table 3:** Incidence rates and hazard ratios of CKD in the urate-lowering therapy for the propensity score matched ULT users and nonusers

	Urate-lowering Therapy									Adjusted HR (95% CI)^*^	*p*-value
	No			Yes							
	No. CKD	Person-years	IR	No. CKD		Person-years		IR			
Urate-lowering therapy	782	45,490	1.7	794		45,799		1.7		0.97 (0.88-1.07)	0.581
XO inhibitors	782	45,490	1.7	296		15,199		1.9		1.07 (0.93-1.22)	0.336
Uricosuric agents	782	45,490	1.7	498		30,600		1.6		0.93 (0.83-1.04)	0.189
**Gender**											
Female	127	6,151	2.1	147		6,864		2.1		0.95 (0.74-1.21)	0.664
Male	655	39,339	1.7	647		38,934		1.7		0.98 (0.88-1.10)	0.759
**Age, year**											
20-39	112	14,677	0.8	126		15,253		0.8		1.09 (0.84-1.41)	0.53
40-59	359	22,213	1.6	294		20,040		1.5		0.92 (0.78-1.07)	0.263
60-79	311	8,600	3.6	374		10,505		3.6		1.00 (0.85-1.16)	0.951
**CCI**											
0	600	40,384	1.5	632		40,923		1.5		1.00 (0.89-1.12)	0.979
1	135	3,994	3.4	103		3,527		2.9		0.83 (0.64-1.08)	0.169
2	47	1,111	4.2	59		1,349		4.4		1.00 (0.67-1.48)	0.989
**HT/DM**											
No/No	702	42,911	1.6	717		43,064		1.7		0.98 (0.88-1.09)	0.705
Yes/No	44	1,784	2.5	50		1,794		2.8		1.07 (0.70-1.63)	0.746
No/Yes	34	745	4.6	22		890		2.5		0.52 (0.28-0.97)	0.04
Yes/Yes	2	50	4.0	5		50		10.0		1.52 (0.20-11.29)	0.684
**Hyperlipidemia**											
No	741	42,861	1.7	747		43,240		1.7		0.97 (0.87-1.07)	0.523
Yes	41	2,628	1.6	47		2,559		1.8		1.06 (0.67-1.67)	0.807
**CVD**											
No	763	44,933	1.7	775		45,341		1.7		0.97 (0.88-1.08)	0.581
Yes	19	557	3.4	19		457		4.2		0.96 (0.45-2.06)	0.921
**ACEI/ARBs**											
No	596	39,795	1.5	612		40,272		1.5		0.99 (0.89-1.11)	0.891
Yes	186	5,695	3.3	182		5,526		3.3		0.92 (0.74-1.13)	0.419
**Diuretics**											
No	626	40,754	1.5	635		41,180		1.5		0.97 (0.87-1.09)	0.635
Yes	156	4,736	3.3	159		4,619		3.4		1.01 (0.80-1.26)	0.941
**Duration from gout diagnosis to the urate lowering therapy, year**											
< 0.5	98	5,835	1.7		563		32,547		1.7	0.96 (0.77-1.19)	0.708
≥ 0.5	684	39,655	1.7		231		13,251		1.7	1.03 (0.88-1.20)	0.719
											

IR: incidence rate (per 100 person-years); HR: hazard ratio; XO inhibitors: xanthine oxidase inhibitors (e.g., allopurinol and febuxostat); Uricosuric agents (e.g., benzbromarone, probenecid, and sulfinpyrazone); DM: diabetes mellitus; CAD: coronary artery disease; CVD: cerebrovascular disease; CCI: Charlson comorbidity index; ACEI: angiotensin-converting-enzyme inhibitor; ARBs: angiotensin-receptor blockers. *Adjusted for sex, age, comorbidities, CCI and medications listed in Table 1.

**Table 4:** Multivariable Cox regression model for the association between urate-lowering therapy and CKD development

Urate-lowering therapy (follow-up period)	Adjusted HR (95% CI)^*^	*p*-value	Adjusted HR (95% CI)^*^	*p*-value
Nonuser	Reference		0.85 (0.71-1.02)	0.073
XO inhibitors only	1.18 (0.98-1.41)	0.073	Reference	
Uricosuric agents only	0.96 (0.84-1.09)	0.493	0.81 (0.67-0.99)	0.037
Both	0.91 (0.80-1.04)	0.187	0.78 (0.64-0.95)	0.013

IR: incidence rate; HR: hazard ratio; XO inhibitors: xanthine oxidase inhibitors (e.g., allopurinol and febuxostat); Uricosuric agents (e.g., benzbromarone, probenecid, and sulfinpyrazone). *Adjusted for sex, age, comorbidities, CCI and medications listed in Table 1.
